# Assessment of hypoxia status in a rat chronic liver disease model using IVIM and T1 mapping

**DOI:** 10.3389/fmed.2024.1477685

**Published:** 2025-01-21

**Authors:** Wenlu Dong, Longyang Xiao, Ziwei Luo, Haiyang Yu, Lili Wang, Yuanxiang Gao, Zhiming Li

**Affiliations:** ^1^Department of Radiology, The Affiliated Hospital of Qingdao University, Qingdao, China; ^2^Department of Pathology, The Affiliated Hospital of Qingdao University, Qingdao, China

**Keywords:** hypoxia, chronic liver disease, IVIM, T1 mapping, pimonidazole, HIF-1α

## Abstract

**Objectives:**

This study was aimed to assess the diagnostic performance of intravoxel incoherent motion (IVIM) magnetic resonance imaging (MRI) and T1 mapping in detecting hypoxia status of chronic liver disease using a carbon tetrachloride (CCl_4_)-induced rat model.

**Materials and methods:**

The hypoxia group of chronic liver disease consisted of eight rats induced by injection of CCl_4_ and the control group consisted of nine rats injected with pure olive oil. All 17 rats underwent MRI examination at week 13 after injection, using T1 mapping and IVIM. Liver specimens were subjected to immunohistochemical staining for the exogenous hypoxia marker pimonidazole and the endogenous hypoxia marker HIF-1α and scored semi-quantitatively. Differences in MRI multiparameters, pimonidazole H-scores, and HIF-1α were analyzed between the control and hypoxia groups. Correlations between MRI multiparameters and H-score, and MRI multiparameters and HIF-1α, were analyzed, and the diagnostic performance of multiparameter MRI was evaluated by receiver operating characteristic (ROC) curve analysis.

**Results:**

There were significant differences between the control group and the hypoxia group in D* values (*p* = 0.01) and *f* values (*p* = 0.025) of IVIM parameters, T1 mapping (*p* = 0.003), HIF-1α (*p* < 0.001) and pimonidazole scores (*p* = 0.004). D* (*r* = 0.508, *p* = 0.037) and T1 mapping (*r* = 0.489, *p* = 0.046) values positively correlated with pimonidazole scores. D* (*r* = 0.556, *p* = 0.020) and T1 mapping (*r* = 0.505, *p* = 0.039) showed a positive correlation with HIF-1α. The optimal cut-off value of T1 mapping was 941.527, and the sensitivity, specificity, and AUC were 87.5, 77.8, and 0.889 (95% confidence interval [CI]: 0.734–1), respectively.

**Conclusion:**

IVIM and T1 Mapping are promising methods for non-invasive detection of hypoxia status in chronic liver diseases.

## Introduction

Chronic liver disease (CLD) is a significant public health burden worldwide, causing over 2 million deaths annually (1). Although several causes behind the development of CLD, some of the most common include alcohol abuse, obesity/metabolic disease, autoimmune hepatitis, and viral hepatitis (hepatitis B virus [HBV] and hepatitis B virus [HCV]) (2). The course of chronic liver disease (CLD) is marked by a prolonged history of chronic parenchymal injury, persistent activation of the inflammatory response, and continuous activation of hepatic fibrosis and wound healing responses (3).

Chronic liver diseases, such as metabolic fatty liver disease (MAFLD) and viral hepatitis, are characterized by persistent inflammation and subsequent liver fibrosis (4, 5). Metabolic fatty liver disease (MAFLD) is a recent terminology that refers to non-alcoholic fatty liver disease (NAFLD)—more precisely denotes the metabolic dysfunction that primarily drives the condition and highlights the pivotal role of metabolic dysfunction in the progression of liver disease. Improvements in nomenclature, diagnostic criteria, heterogeneity, and subtype research will facilitate comprehensive studies on MAFLD and yield more effective therapy and care approaches for patients (6). Liver fibrosis is a dynamic, highly integrated process in chronic liver injury of any etiology and is characterized by an abnormal accumulation of extracellular matrix (ECM) (7). The progression of liver fibrosis can lead to liver cirrhosis and even to hepatocellular carcinoma (HCC). The advancement of hepatocellular carcinoma is intricately linked to liver fibrosis, with over 80% of hepatocellular carcinomas (HCC) manifesting in fibrotic or cirrhotic livers (8). This indicates that liver fibrosis significantly contributes to the liver’s precancerous environment (PME) (9). Liver fibrogenesis and the chronic inflammatory response, play a primary role in the progression of chronic liver diseases (CLD) (5). In addition, circadian rhythms significantly influence the advancement of metabolic disorders, including NAFLD, with numerous genes associated with metabolic regulation governed by these rhythms. Circadian disruption may influence the initiation and progression of NAFLD/non-alcoholic steatohepatitis (NASH), expedite the fibrotic response, and eventually result in HCC. Connecting biological clocks to metabolic processes and carcinogenesis offers potential avenues for treating and preventing metabolic liver disease and hepatocellular carcinoma (HCC) (10–12). Hepatic hypoxia plays a vital role in the formation and progression of chronic liver disease (13, 14). Hepatic hypoxia is present at all stages of chronic liver disease and progressively increases with different stages, from early hepatocellular injury to cirrhosis (15). Mesarwi et al. (16) found that hypoxia accelerated the progression of non-alcoholic fatty liver disease (NAFLD) and aggravated liver fibrosis. Liver fibrosis, in turn, can significantly aggravate the degree of hypoxia (17). Hypoxia is still the main driver of sustained progression of liver fibrosis (18–20). Chronic liver disease caused by viral infection, excessive alcohol consumption, or metabolic disorders all activate HIF through hypoxia-dependent and hypoxia-independent signaling (13). HIF-1α is a marker of hepatic hypoxia and produces prohepatic fibrogenic effects mediated by HIF-1α in different cell types within the liver (21). Detecting or monitoring the hypoxia status of chronic liver disease will contribute to a better understanding of the process and finding more proper ways to treat it (22–24).

There is a pressing need for a non-invasive and accurate method for assessing hepatic hypoxia for clinical practice. Intravoxel incoherent motion (IVIM) MRI is a functional imaging technique that utilizes the Brownian motion of water molecules, and its parameters can reflect tissue diffusivity and tissue microcapillary perfusion (25, 26). Previous studies have applied IVIM to monitor hypoxia in different types of malignant tumors (27–29). Malignant tumors are frequently linked to reduced oxygen levels in the tumor tissue. The hypoxic environment may facilitate the emergence of distant metastases and adversely affect patient prognoses (30). The prognostic relevance of HIF-1α expression to various malignant tumors has been demonstrated (31–33). Consequently, the assessment of tumor hypoxia using IVIM magnetic resonance imaging (MRI) is crucial for prognostic evaluation and treatment decision-making. However, limited studies exist about the application of IVIM for identifying hypoxic conditions in chronic liver disease. MR T1 mapping technique is a non-invasive quantitative method to reflect the T1 relaxation time of specific tissue. T1 relaxation time is prolonged in liver fibrosis due to excessive ECM deposition (34). At present, T1 mapping for the evaluation of liver fibrosis has been widely studied (35). However, a few studies have reported detecting the hypoxia status of chronic liver disease using the IVIM and T1 mapping.

In this study, therefore, we proposed to apply IVIM and T1 mapping for detecting the hypoxia status of chronic liver disease in a CCl_4_-induced rat model.

## Materials and methods

The Institutional Animal Care and Welfare Committee of our institution approved this study.

### Chronic liver diseases rat model

In total, 17 healthy Wistar male rats (250 ± 10 g, 6 weeks old) were randomly divided into two groups, namely, a hypoxia group of chronic liver disease (*n* = 8) and a control group (*n* = 9). All Wistar Rats were housed in a specific pathogen-free (SPF) animal house with environmental conditions of 21 ± 2°C, humidity (52.5 ± 12.5)%, and a light–dark cycle of 12 h. All rats started the experiments 1 week after acclimatization when they had free access to water and commercial rat chow (Meadow Feeds™ Standard Maintenance Rat Chow, Keao Xieli Feed Co., Ltd, Tianjin, China). The rats in the hypoxia group of chronic liver disease were subjected to intraperitoneal (i.p.) injection of 3 mL/kg CCl_4_ dissolved in olive oil, with a ratio of 4:6, twice per week at 1–6 weeks and once per week at 7–13 week. Whereas the rats in the control group underwent intraperitoneal injections of 3 mL/kg of pure olive oil solution similarly. Changes in the body weight of rats in the hypoxia and control groups were analyzed over 13 weeks to determine their health status.

### MRI examination

At week 13 after the initial injection of CCl_4_ and oil, rats in both the hypoxia group (*n* = 8) and control group (*n* = 9) underwent MRI examinations with an 8-channel coil (CG-MUC22-H300-AG, Chenguang, Shanghai, China) on a 3-T MRI scanner (Magnetom skyra, Siemens Medical Solutions Erlangen, Germany). All rats were injected with pimonidazole (Hypoxyprobe-3; Hypoxyprobe, Inc., Burlington, MA, USA) through the tail vein with a dose of 60 mg/kg before MR scanning. Furthermore, the rats were placed in a small animal anesthesia equipment by ventilation anesthesia with 3.5% isoflurane (RWD Life Science Co., Ltd, Shenzhen, China). T1 mapping was performed using the variable flip angle (VFA) T1 mapping technique, flip angle 1 is 3°, and flip angle 2 is 17°. The parameters were as follows: Three orthogonal directions: time of repeat (TR), 5.48 ms; time of echo (TE), 19 ms; field of view (FOV), 100 mm × 100 mm; slice thickness, 2.0 mm, 20 slices. IVIM was acquired using a free-breathing single-shot echo-planar imaging pulse sequence with eight *b*-values. The parameters were as follows: TR, 4,900 ms; TE, 64 ms; FOV, 136 mm × 136 mm; slice thickness, 2.5 mm; 20 slices; *b* = 0, 50, 100, 150, 200, 400, 800, and 1,200 mm^2^/s; scan time, 10 min.

### Image analysis

T1 maps, and *f*, D, and D* maps, were collected for image analysis. To reduce the error caused by the small sample size, we outlined three releases of information (ROIs) at the maximum level of each slice, excluding artifacts, significant vessels, bile ducts, and liver boundaries, and the average value was used as the final measurement result. The area of ROI was controlled within 6–8 cm^2^. The IVIM data were postprocessed using Medical Imaging Interaction Toolkit (MITK)-Diffusion software (MITKv2023.04, German Cancer Research Center, Heidelberg, Germany). The IVIM mode is a biexponential model defined by the parameters *f*, D, and D*. The fitting method we use is Fit D & f (high *b*), then fit D*: First fit *f* and D (monoexponentially (1 − *f*)*e*^{−*bD*}^) and use these; second fixed parameters in a second fit of D* with the complete biexponential model; the equation is expressed as follows:


$$SI/{SI}_{0}=\left(1f\right)\;\exp.\left(bD\right)+f\;\exp.\left({bD}^{*}\right)$$

where SI_0_ denotes the mean signal intensity of the ROI under consideration at *b* = 0 mm^2^/s, and SI represents the signal intensity at higher *b* values, D* refers to the microcirculation perfusion (pseudo-diffusion) coefficient, *f* is the perfusion fraction, and f multiplied by D* is the perfusion coefficient, D—also called the slow diffusion coefficient—which is non-perfusion-related but molecular diffusion-related diffusivity, represents the actual molecular diffusion (36). This equation was established as an IVIM-DWI exponential model. The T1 mapping data were postprocessed using Syngovia software on an Advantage Workstation (Version VB20A, Siemens Medical Solutions Erlangen, Germany). The mean values of T1 relaxation time, D, D*, and *f* for each ROI were generated.

### Histologic evaluation

After MR scanning, the rats were euthanized immediately, and then the liver tissues were harvested. Liver specimens were fixed in formalin, embedded in paraffin, and sectioned. Some sections were stained with hematoxylin–eosin (HE) for morphological analysis of liver parenchyma, and some sections were stained with Masson staining and immunohistochemical staining for *α*-smooth muscle actin (α-SMA) to assess the extent of liver fibrosis. The remaining fixed liver tissues were subjected to immunohistochemical staining for the endogenous hypoxia marker, hypoxia-inducible factor-1α (HIF-1α), and the exogenous hypoxia marker, pimonidazole. The histopathologic results were determined by two pathologists with more than 10 years of experience in liver pathology. The degree of liver fibrosis was evaluated using the meta-analysis of histological data in viral hepatitis (METAVIR) scoring system and was divided into five stages from F0 to F4. The area of Masson positive areas was measured using image analysis software (Image J, version 1.52a, National Institute of Health, Bethesda, Maryland 20892 USA).

### Immunohistochemical staining

Paraffin sections were taken as antigen, closed with 5% BSA, and then incubated with primary antibody a-SMA (Rabbit, 14395-1-AP, Proteintech, USA) overnight. Sections were incubated with secondary antibody at 37°C for 20 min, stained with DAB, counterstained with hematoxylin, and observed under a light microscope, and the integrated optical density (IOD) was measured by Image-Pro Plus software to determine the expression abundance. After antigen repair, the sections were incubated with anti-HIF-1α antibody (HIF1-α, PB9253, Boster, Wuhan, China) at 4°C overnight. Sections were then incubated with secondary antibody (1:200 dilution) for 2 h at RT and re-stained for 30 s. The area of HIF-1α-positive regions was analyzed semi-quantitatively using ImageJ open-source software (Image J, version 1.52a, National Institute of Health, Bethesda, Maryland 20892 USA). Sections were incubated with primary antibody pimonidazole (Hypoxyprobe, Inc., Burlington, MA, USA) for 30 min. Pimonidazole staining was visualized with 3,3-diaminobenzidine (DAB), and sections were restained with hematoxylin and scored semi-quantitatively with H-score. The H-score—including the percent of positive pimonidazole cells and coloring strength—for all the rectangular ROIs was automatically evaluated by software using (3DHISTECH Ltd., Budapest, Hungary) (37).


$$\begin{array}{c} H-\mathrm{score}=\sum \left(\mathrm{pi}\times i\right)=\left(\mathrm{percentage of weak intensity}\times 1\right)\\ +\left(\mathrm{percentage of moderate intensity}\times 2\right)\\ +\left(\mathrm{percentage of strong intensity}\times 3\right) \end{array}$$

Where pi denotes the positive signal pixel area/number of cells as a percentage and i represents the intensity of coloring (38). The H-score measures how much of a specific biomarker is present in an immunohistochemistry (IHC) image. It ranges from 0 to 300, with higher values indicating a more severe hypoxia (39).

### Statistical analysis

All statistical analyses were performed using the Statistical Package for the Social Sciences (SPSS) version 25.0 (IBM). The Kolmogorov–Smirnov test was utilized to evaluate the normality of the data. The IVIM parameters (D, f, and D*) and T1 mapping demonstrated a normal distribution. A one-way ANOVA was utilized to do multiple comparisons of IVIM parameters and T1 mapping. Multiple two-by-two comparisons were conducted employing Bonferroni correction. Spearman’s correlation coefficient was used to assess the correlation between different imaging parameters (D, D*, *f*, and T1 relaxation time) and the H-score, and the correlation between different imaging parameters and the HIF-1α semiquantitative score. Simple linear regression analyses were performed to illustrate the correlation between imaging parameters and H-score, imaging parameters, and HIF-1α semiquantitative score. The differences between the control group and the hypoxia group were assessed. An Independent sample *t*-test was used for continuous variables. The *p* < 0.05 were considered statistically significant. Receiver operating characteristic (ROC) curve analysis was performed, and sensitivity and specificity were calculated. The areas under the ROC curve (AUCs) were compared to evaluate the diagnostic performance of IVIM and T1 mapping.

## Results

### Animal model and histopathological observations

In total, 17 rats underwent histopathological examination. According to HE staining, there were nine rats with no fibrosis, one rat with fibrosis stage F1, two rats with F2, one rat with F3, and four rats with F4. We sent 17 paraffin blocks for HIF-1*α* and α-SMA immunohistochemical staining, and 1 paraffin block from the experimental group was missing during the experiment. To maintain the integrity of the dataset, we used the mean HIF-1*α* values and α-SMA values of the remaining seven rats in the experimental group to represent the missing values. The integrated optical density (IOD) of α-SMA in the hypoxia group was higher than that in the control group (45130.692 ± 22212.153 vs. 742.720 ± 375.388, *p* < 0.001). The percentage area of positive Masson immunohistochemical staining was statistically significantly different between the control and hypoxia groups (9.142 ± 3.542 vs. 0.253 ± 0.068, *p* < 0.001) (Table 1). These rats were divided into three groups based on histopathological findings, including the group with no fibrosis (F0, *n* = 9), the group with progressive fibrosis (F1–F3, *n* = 4), and the group with severe fibrosis or cirrhosis (F4, *n* = 4). A one-way ANOVA was used to test whether there was a dose–response relationship between the severity of liver injury and imaging findings. The results showed a statistically significant trend of increasing D* and T1 mapping values with increasing fibrosis stage (Table 2). Table 3 shows that D* values were statistically different in the progressive liver fibrosis and no liver fibrosis groups, and T1 mapping was statistically different in the no liver fibrosis and liver cirrhosis groups.

**Table 1:** Mean parameter values for pathological and imaging features.

Imaging/pathological parameter	Control group (mean ± SD, *N* = 9)	Hypoxia group of CLD (mean ± SD, *N* = 8)	*p*
T1 mapping	920.860 ± 35.011	990.798 ± 46.632	**0.003**
D*(×10^−6^ mm^2^/s)	0.051 ± 0.008	0.064 ± 0.011	**0.01**
*f* (%)	0.342 ± 0.595	0.274 ± 0.523	**0.025**
D (×10^−6^ mm^2^/s)	0.001 ± 0.002	0.001 ± 0.002	0.72
H-SCORE	113.427 ± 3.152	118.650 ± 3.462	**0.005**
HIF-1α (area %)	17.173 ± 6.364	29.763 ± 3.801	**<0.001**
α-SMA (IOD)	742.720 ± 375.388	45,130.692 ± 22212.153	**<0.001**
Masson (area %)	0.253 ± 0.068	9.142 ± 3.542	**<0.001**
Weight (g)	357.017 ± 18.734	310.161 ± 23.629	**<0.001**

CLD, chronic liver disease; SD, standard deviation. Bold values mean that *p* < 0.05 was considered statistically significant.

**Table 2:** Pure molecular diffusion (D), perfusion fraction (*f*), microcirculation perfusion coefficient (D*), and T1 mapping values in different stages of fibrotic livers.

Rats	T1 mapping	D* (×10^−6^ mm^2^/s)	D (×10^−6^ mm^2^/s)	*f*
No liver fibrosis group (*n* = 9)	920.860 ± 35.011^†^	0.051 ± 0.008	0.001 ± 0.0002	0.342 ± 0.060
Progressive liver fibrosis group (*n* = 4)	970.970 ± 47.589	0.066 ± 0.012^†^	0.001 ± 0.0001	0.268 ± 0.048
liver cirrhosis group (*n* = 4)	1010.625 ± 41.964^†^	0.063 ± 0.010^†^	0.001 ± 0.0001	0.279 ± 0.063
F	7.633	4.207	2.325	2.975
*p*-value	0.006	0.037	0.134	0.084

^†p < 0.05 Comparing liver cirrhosis group or progressive liver fibrosis group with no liver fibrosis group, with Bonferroni adjustment for multiple comparisons.^

**Table 3:** The *p*-value with Bonferroni adjustment for comparison of pure molecular diffusion (D), perfusion fraction (*f*), microcirculation perfusion coefficient (D*), and T1 mapping values in different stages of fibrotic livers.

	T1 mapping	D* (×10^−6^ mm^2^/s)	D (×10^−6^ mm^2^/s)	*f*
No liver fibrosis vs. progressive liver fibrosis group	0.053	**0.020**	0.376	0.051
No liver fibrosis vs. liver cirrhosis group	**0.002**	0.066	0.137	0.096
Progressive liver fibrosis vs. liver cirrhosis group	0.178	0.608	0.053	0.775

Bold values indicate *p* < 0.05, which is statistically significant.

**Figure 1:**
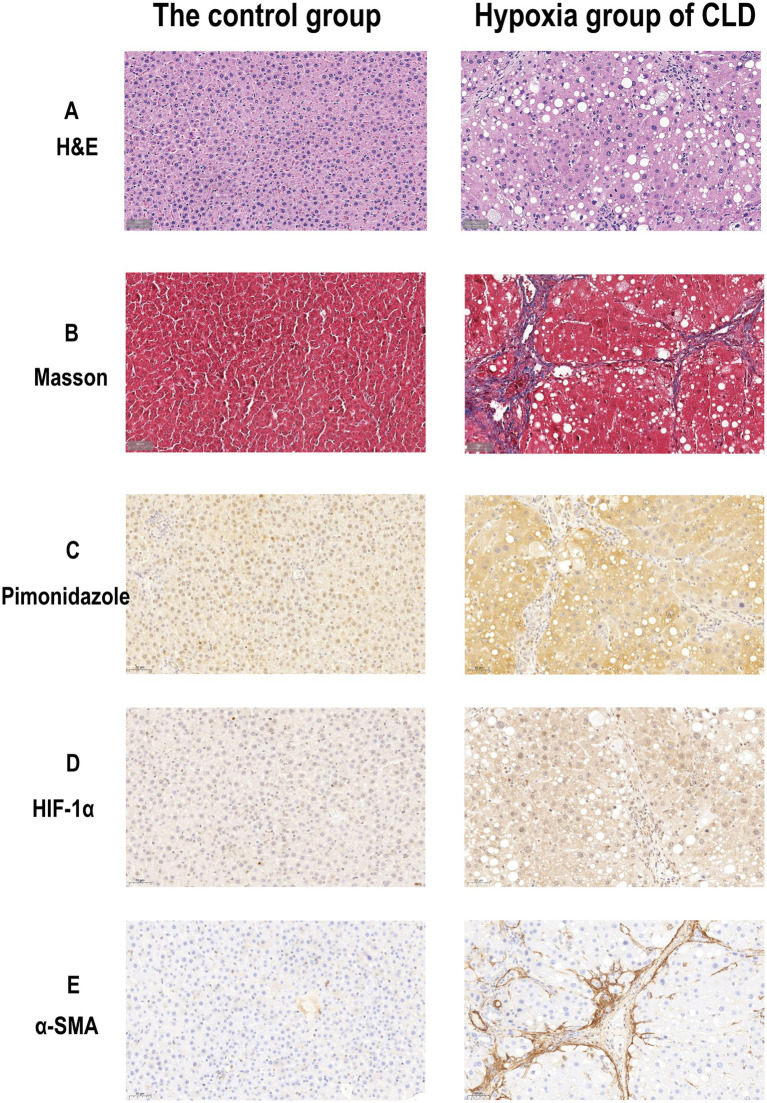
Histological and immunohistochemical images of the rat livers of the control group and hypoxia group of CLD. **(A)** H&E staining images (× 40) of the control and hypoxia group of CLD. **(B)** Masson staining images (× 40) of the control group and hypoxia group of CLD. **(C)** Pimonidazole Immunohistochemical staining images (× 40) of control and hypoxia group of CLD. **(D)** HIF-1α Immunohistochemical staining images (× 40) of control and hypoxia group of CLD. **(E)** α-SMA Immunohistochemical staining images (× 40) of control and hypoxia group of CLD.

The H-score in the hypoxia group was higher than that in the control group (118.650 ± 3.462 vs. 113.427 ± 3.152, *p* = 0.005). The HIF-1α semiquantitative score (area%) in the hypoxia group was higher than that in the control group (29.763 ± 3.801 vs. 17.173 ± 6.364, *p* < 0.001) (Table 1). The examples of histological and immunohistochemical images of the control and hypoxia groups are shown in Figure 1. The average weight in the hypoxia group of CLD was lower than that in the control group (310.161 ± 23.629 vs. 357.017 ± 18.734, *p* < 0.001). With the progression of liver fibrosis, the average body weight of the rats gradually decreased; the weight in the liver cirrhosis group was lower than the progressive liver fibrosis group (330.01 ± 24.271 vs. 293.180 ± 4.880, *p* = 0.025).

### Descriptive analysis of MRI parameters

**Figure 2:**
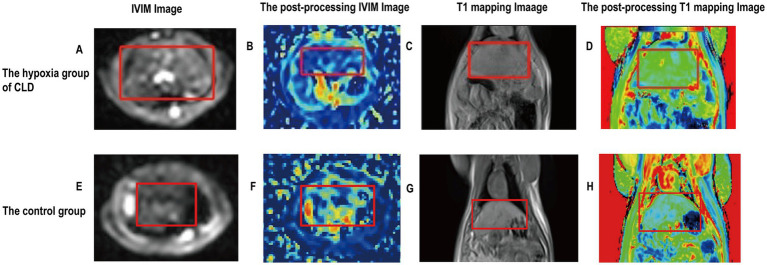
Imaging images of the rat livers of the control group and hypoxia group of CLD. **(A)** Rat liver fibrosis was observed in an IVIM image. **(B)** The postprocessing IVIM of liver fibrosis; D* value is 0.055. **(C)** Rat liver fibrosis was observed in a T1WI image. **(D)** The postprocessing T1 mapping of liver fibrosis; T1 mapping value is 992.191. **(E)** Rat liver was observed in an IVIM image. **(F)** The postprocessing IVIM of the liver; D* value is 0.042. **(G)** The rat liver was observed in a T1WI image. **(H)** The postprocessing T1 mapping of the liver; T1 mapping value is 880.460. Rat livers are shown in red labeled boxes.

The *f* value of the hypoxia group was lower than those of the control group (0.274 ± 0.523 vs. 0.342 ± 0.595, *p* = 0.025). The D* and T1 relaxation time of the hypoxia group were higher than those of the control group (0.064 ± 0.011 vs. 0.051 ± 0.008, *p* = 0.01; 990.798 ± 46.632 vs. 920.860 ± 35.011, *p* = 0.003). There was no significant difference in D values between the hypoxia group and the control group (Table 1). The examples of MR images of the control group and hypoxia group are shown in Figure 2.

### Correlation between MRI parameters and H-score, MRI parameters, and HIF-1α

**Figure 3:**
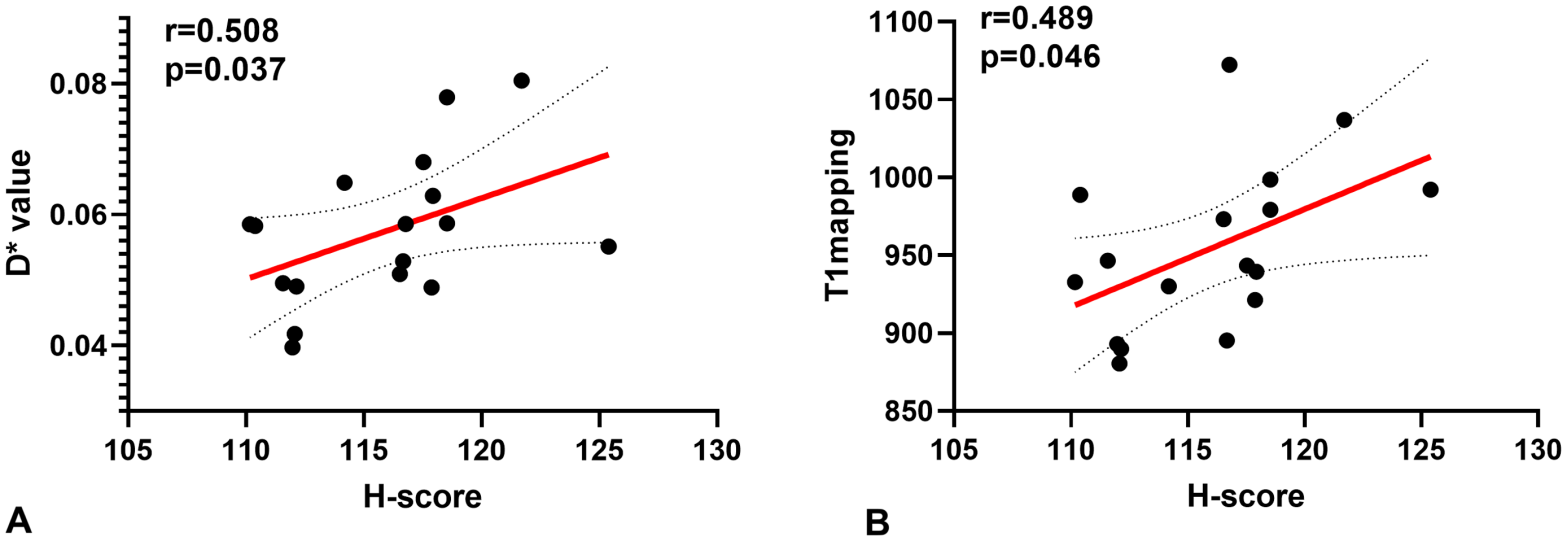
Correlations for the hypoxia score (H-score) with the pseudo diffusion coefficient (D*) and the T1 mapping. **(A)** H-score correlated positively with the D* (*r* = 0.508, *p* = 0.037). **(B)** H-score correlated positively with T1 mapping (*r* = 0.489, *p* = 0.046).

**Figure 4:**
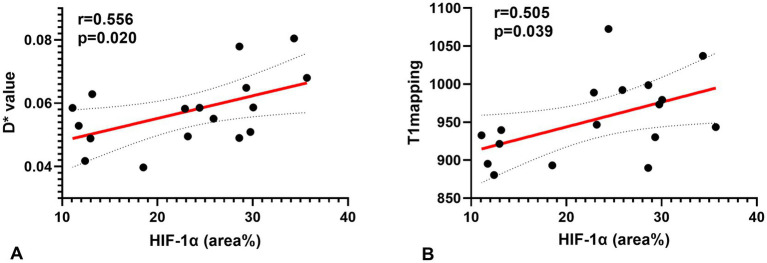
Correlations for the hypoxia score (HIF-1α) with the pseudo-diffusion coefficient (D*) and the T1 mapping. **(A)** HIF-1α correlated positively with the D* (*r* = 0.556, *p* = 0.020). **(B)** HIF-1α correlated positively with T1 mapping (*r* = 0.505, *p* = 0.039).

**Figure 5:**
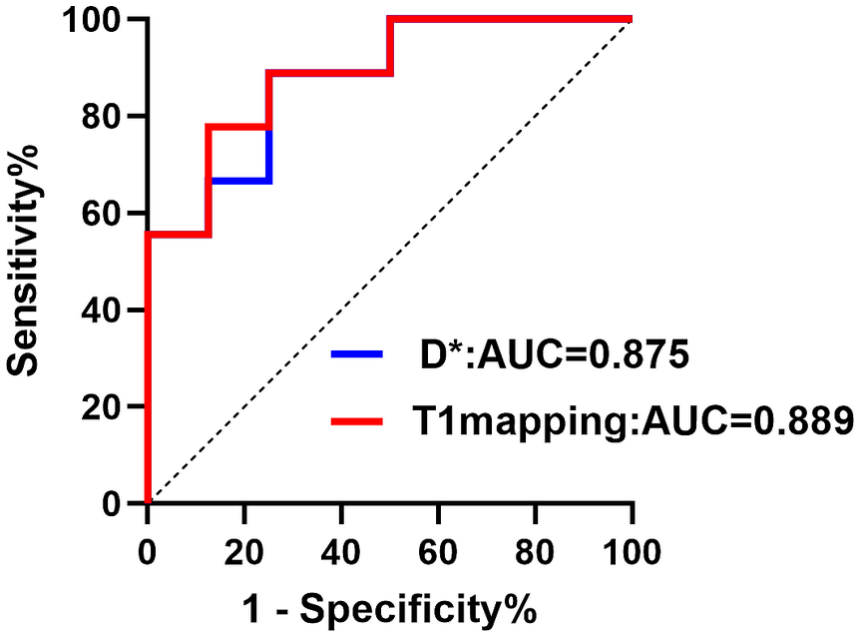
The ROC curve showed the performance of D* and T1 mapping in predicting hypoxia. Receiver operating characteristic (ROC) curves for D*, T1 mapping. The AUC values were 0.875 and 0.889, respectively.

A Spearman correlation analysis revealed no correlation between D and the H-score (*r* = 0.15, *p* = 0.573), *f* and the H-score (*r* = −0.206, *p* = 0.330). The H-score had a moderate correlation with D* (*r* = 0.508, *p* = 0.037) and a weak correlation with T1 mapping (*r* = 0.489, *p* = 0.046) (Figure 3). A Spearman correlation analysis revealed no correlation between D and the HIF-1α (*r* = 0.066, *p* = 0.801), *f* and the HIF-1α (*r* = −0.240, *p* = 0.353). The HIF-1α had a moderate correlation with D* (*r* = 0.556, *p* = 0.020); and a moderate correlation with T1 mapping (*r* = 0.505, *p* = 0.039) (Figure 4). The hypoxia group and the control group were differentiated with the cut-off value D* = 0.059, and the sensitivity, specificity, and 95% CI of D* as 75%; 88.9%; and 0.71, 1, respectively. The cut-off value of T1 mapping was 941.527, and the sensitivity, specificity, and 95% CI were 87.5%; 77.8%; and 0.734, 1, respectively. The AUCs of D* and T1 mapping for differentiating the hypoxia group and control group were 0.875 and 0.889 (Figure 5).

## Discussion

Our study used IVIM and T1 mapping to detect chronic liver disease hypoxia status. The results showed that the parameters of D* and T1 mapping were significantly correlated with the H-score of pimonidazole and HIF-1α. Furthermore, D* was the most reliable parameter to evaluate hypoxia status in chronic liver disease.

CLD is a highly dynamic condition. Chronic liver injury is known to lead to the persistent activation of liver fibrogenesis, which is a critical and dynamic biological process involving numerous molecular mechanisms, mediators, and interactions/responses of different cell populations in liver fibrosis (40–42). Liver fibrosis is regarded as a potentially reversible pathophysiological event characterized by excess deposition of extracellular matrix (ECM) components (43). Hypoxia plays a vital role in this dynamic process (44), and it may also be a potential target for the treatment of chronic liver disease (45). Pathologically, hypoxia status can be detected by pimonidazole staining (46, 47). We quantitatively analyzed immunohistochemical images using QuantCenter software to calculate a density heatmap of pimonidazole, including quantitatively determining the staining intensity and the corresponding percentage of positive cells. The automatic calculation of H-scores prevents the introduction of bias from different pathologists calculating H-scores (48). We also used an additional hypoxia marker, HIF-1α, to further assess and validate hypoxia in the liver. HIF-1α is a transcription factor mediating cellular and systemic homeostatic responses to reduced oxygen supply in mammals, including angiogenesis, erythropoiesis, and glycolysis. HIF-1α directly regulates cell survival and function in the inflammatory microenvironment and plays a key role in cellular adaptation to changes in oxygen supply (49–51). The study results showed a statistically significant difference in oxygen concentration between the chronic liver disease group and the control group, whether measured by HIF-1α or pimonidazole.

Many previous studies have demonstrated that IVIM can be used to detect hypoxia in different types of tumors (27–29). For predicting hypoxia niches in glioma, the D* value showed a negative correlation with HIF-1α expression. A dysfunctional and impaired vasculature in gliomas reduces blood and oxygen supply, decreasing D* values, while HIF-1α expression increases as tumor cells grow (52). Moreover, the D* and *f* values of IVIM are reliable parameters for predicting the expression of HIF-1α in soft tissue sarcomas (28). The results of our study also showed that the IVIM parameters could be used to detect the hypoxic status of liver fibrosis. The positive correlation between D* and H-score, D* and HIF-1α could be attributed to the theory of D* mainly being a measure of microcirculation perfusion. The neovascularization in the liver was found to be directly proportional to the degree of hepatic fibrosis (53). Hypoxia is one of the main drivers of angiogenesis. Studies have shown that hepatocyte hypoxia and angiogenesis progress together with fiber formation after liver injury (15). Due to the increase of hepatic neovascularization during hypoxia status, there may be a corresponding increase in local blood microperfusion, resulting in increased D*. However, our study showed that D and *f* cannot be used as parameters to identify hypoxic status in chronic liver disease, because of technical problems, such as data instability and a low SNR, which resulted in inaccurate measurement of *f* and D. These factors may be the main reasons why these factors did not work well in predicting low oxygen levels in chronic liver disease (54).

The T1 mapping technique is a noninvasive quantitative method for evaluating the T1 relaxation time of liver tissues. The T1 value can quantitatively assess the content of water molecules and macromolecules in the tissues (55). Excess extracellular matrix (ECM) results in extended T1 relaxation, thus, T1 mapping can effectively evaluate liver fibrosis stages (56, 57). Our study concluded that T1 mapping can assess the hypoxic status of chronic liver disease. We found that the H-score—an indicator of hepatic hypoxia—positively correlated with T1 mapping values. The MRI longitudinal relaxation rate R1 (1/T1) is sensitive to changes in the level of molecular oxygen (O_2_) dissolved in plasma or interstitial tissue fluids (58–60). In hypoxic tissues, the decrease in dissolved oxygen, due to the paramagnetic properties of dissolved oxygen, leads to a reduction in the tissue’s R1 rate, which means an increase in T1 time. The detection of hypoxic zones is standard at all stages of chronic liver disease and increases progressively from early injury to the development of cirrhosis (54). The above reasons may explain why T1 values positively correlate with the degree of hypoxia.

Our study has several limitations. First, the sample size of this study was relatively small and we could not confirm whether there were differences in the level of hypoxia in the liver parenchyma of rats with different stages of chronic liver disease. We will increase the sample size and continue to improve the experiment. Second, the experimental design was inadequate, as it lacked the administration of antioxidants and the targeting of metabolic pathways involved in hypoxia. Furthermore, liver function tests and oxidative stress tests were not conducted. In subsequent research, these experiments will be incorporated. Third, the ROIs need to be selected manually to avoid necrotic areas, which inevitably introduce errors. Fourth, we will use intelligent software for automatic sketching to reduce the error. In addition, the occurrence and progression of liver fibrosis in animals is not the same as in humans, and therefore further clinical research in liver fibrosis will be required.

In conclusion, IVIM and T1 Mapping are promising methods for non-invasive detection of hypoxic status in chronic liver disease. The D* and T1 relaxation time are significantly correlated with hypoxia and, therefore, can be used for the assessment of hypoxic status in chronic liver disease. We provide a feasible method for non-invasive hypoxia imaging in chronic liver disease.

## Data Availability

The raw data supporting the conclusions of this article will be made available by the authors, without undue reservation.
